# Recent Advances in the Applications and Studies of Polysaccharide-, Protein-, and Lipid-Based Delivery Systems in Enhancing the Bioavailability of Capsaicin—A Review

**DOI:** 10.3390/polym17091196

**Published:** 2025-04-27

**Authors:** Xiang Qiu, Jing Xie, Jun Mei

**Affiliations:** 1 College of Food Science and Technology, Shanghai Ocean University, Shanghai 201306, China; m240451273@st.shou.edu.cn; 2 National Experimental Teaching Demonstration Center for Food Science and Engineering, Shanghai Ocean University, Shanghai 201306, China; 3 Shanghai Engineering Research Center of Aquatic Product Processing and Preservation, Shanghai Ocean University, Shanghai 201306, China; 4 Shanghai Professional Technology Service Platform on Cold Chain Equipment Performance and Energy Saving Evaluation, Shanghai Ocean University, Shanghai 201306, China

**Keywords:** capsaicin, bioactivity, metabolism, delivery system, food preservation

## Abstract

The primary active ingredient in capsicum is capsaicin. However, capsaicin bioavailability is low due to its restricted water solubility, and its potent spicy flavor will further restrict its use in food. This paper provides a complete overview of capsaicin. The biological activity of capsaicin and its impacts on metabolism in vivo are described. To increase capsaicin stability and bioavailability, several capsaicin-based delivery systems, including liposomes, double emulsions, nanoparticle mesosystems, and multiple systems made of distinct hydrocolloids, are covered in this review. Finally, potential uses for food preservation are introduced in line with this. Numerous delivery systems introduced in this review have effectively solved the problems of poor water solubility and poor bioavailability of capsaicin. Although capsaicin has potential uses in food preservation, there is little research on its application in functional food development. More innovative capsaicin-based delivery methods should be established, and more capsaicin-based applications should be developed in the future.

## 1. Introduction

A well-liked food and spice crop from the Solanaceae family, chili pepper is grown in large quantities in tropical and subtropical climates worldwide [1]. Chili pepper is a great source of a wide range of nutrients and dietary compounds, including capsaicin (CAP), vitamins A and C, pigments, minerals, and essential oils [2].

**Figure 1:**
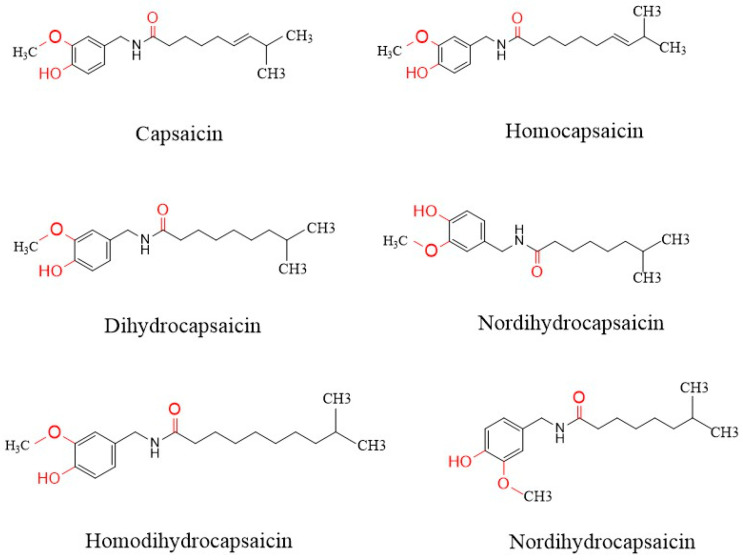
Chemical structures of capsaicinoids.

CAP is the main active component in chili peppers, and its formation mainly consists of two major steps. Firstly, vanillylamine and 8-methylnonene coenzyme A are generated through the phenylpropane metabolic pathway and branched-chain fatty acid pathway. Finally, with the help of CAP synthase, it is formed by the reaction of vanillylamine and 8-methylnonenol coenzyme A in the placental tissue cells of chili peppers [3]. The genus CAP has several types of capsaicinoids, such as CAP, hypercapsaicin, dihydrocapsaicin, hyperdihydrocapsaicin, dehydrocapsaicin, and desmethylcapsaicin (Figure 1). It is the presence of these molecules that gives chili peppers their unique flavor. CAP (*trans*-8-methyl-N-vanillyl-6-nonenamide, C_18_H_27_NO_3_) is a crystalline lipophilic molecule and poorly soluble in water [4]. CAP not only has the ability to improve swallowing dysfunction but also possesses many physiological effects, such as in weight loss, blood lipid regulation, sugar reduction, and anti-fatigue, anti-inflammatory, and anti-tumor properties. But lately, the majority of studies have been on the pharmacological activity, extraction, and purification of CAP. Although CAP is easily absorbed in the human body, its bioavailability is limited by concentration dependence, so its application is also somewhat limited. In response to this situation, researchers have also designed some novel delivery systems to improve the utilization of CAP. CAP also has potential for food preservation due to its antioxidant and antimicrobial activity. More scholars have already used natural active substances for preservation, such as carvacrol [5] for turbot and ginger essential oil [6] for crucian carp. CAP is often added to foods as a natural preservative and antioxidant and is also incorporated into food packaging films and functional food ingredients, providing consumers with a more environmentally friendly and healthier approach to food preservation. In this regard, this paper presents the bioactivity, metabolism, novel delivery systems, and application of CAP in food preservation. The literature search for this review mainly covered the Web of Science Core Collection (2020–2025), Google Scholar (2000–2025), and PubMed (2000–2025). CAP, bioactivity, delivery system, and metabolism were commonly used as keywords to search the literature. The focus was on literature from the last five years and covered the keywords CAP and delivery system.

## 2. Bioactivities of CAP

### 2.1. Analgesic Effect

The mechanism of CAP analgesia primarily involves interaction with pain receptors, which is one of the key molecules in pain perception. When CAP binds to the transient receptor potential cation channel subfamily V member 1 (TRPV1) receptor, it activates the receptor, resulting in the sensation of pain. Different doses and times of CAP produce different effects in the body. A high concentration of CAP activates TRPV1, leading to the ablation of axon endings and long-term de-functioning of TRPV1, ultimately achieving long-lasting analgesia for chronic pain [7]. On the other hand, low concentrations of CAP bind to TRPV1 to desensitize it, lowering its response to endogenous ligands or inflammatory mediators and decreasing nociceptive hypersensitivity [8]. Wang et al. [9] discovered that by ablating the TRPV1+ terminals in the skin, a single subcutaneous local administration of CAP into the skin of the face dramatically decreased trigeminal neuropathic pain in rats for nearly three weeks. Tshering et al. [10] looked into the effects of CAP in people suffering from osteoarthritis (OA). The results showed that topical CAP (0.0125–5%) reduced osteoarthritis pain severity, as determined by an analog scale of vision, and that topical CAP reduced osteoarthritis pain for up to three months. In addition to oral or injectable forms of CAP, many CAP patches are currently used. With relief lasting up to three months, the U.S. Food and Drug Administration formally authorized Averitas Pharma’s Qutenza 8% patch in July 2020 for the alleviation of human foot neural pain brought on by diabetic peripheral neuropathy. Arora et al. [11] further demonstrated that the application of CAP patches was an effective non-opioid analgesic regimen, and 8% topical CAP patches were approved in the European Union for postherpetic neuralgia, trigeminal neuralgia, and for refractory neuralgia that did not respond to other approaches, or for patients who could not tolerate systemic therapy. These CAP patches depleted substance P in peripheral sensory neurons, thus acting as an analgesic. Olusanya et al. [12] compared the analgesic efficacy of the CAP 8% patch (C8P) and CAP 0.025% patch (CON) on focal neuropathic pain caused by spinal cord injury. The C8P was more effective than the CON, reducing pain by 35% and 29% at the 2nd and 4th weeks, respectively. Therefore, the C8P helped individuals with spinal cord injuries and refractory NP with their pain and movement.

Compared with traditional analgesics, CAP has fewer side effects and is an excellent biologic agent, which can improve the analgesic effect and duration when used in combination with analgesics.

### 2.2. Anti-Inflammatory Effect

Chronic inflammation is believed to be the root cause of many chronic diseases, and more than half of all deaths worldwide are directly linked to chronic inflammatory diseases [13]. During inflammation, inflammatory cells are activated to release high levels of a range of cytokines that facilitate the inflammatory response. These cytokines are implicated in the development of numerous diseases, in addition to having the ability to cause tissue damage. It has been discovered that CAP can effectively reduce the inflammatory response of macrophages induced by lipopolysaccharide by inhibiting the nuclear factor κB (NF-κB) and microtubule-associated protein kinase (MAPK) signaling pathways. This could reduce the discharge of inflammatory factors, interleukin, tumor necrosis factor-α (TNF-α), nitric oxide (NO), and other inflammatory mediators [14]. Zhao et al. [15] demonstrated that pretreatment with CAP (100 μM) significantly reduced the inflammatory response triggered by lipopolysaccharide (LPS) via the toll-like receptor 4 (TLR4)/NF-κB signaling pathway, thereby enhancing glucose uptake and barrier integrity. The findings demonstrated that dihydrocapsaicin could successfully reduce the revitalization of NF-κB and its molecular targets in endothelial cells mediated by TNF-α, and dihydrocapsaicin pretreatment could substantially decrease monocyte adhesion to endothelial cell surfaces and promote the production of NO, thus promoting vascular health and inhibiting the inflammatory response of endothelial cells [16].

**Figure 2:**
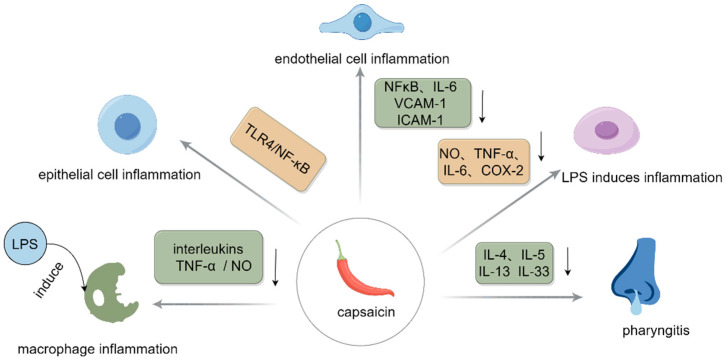
Anti-inflammatory mechanism of CAP. The gray gradient arrows in the figure indicate process arrows and the black solid arrows indicate decreases.

Studies have found that CAP has a synergistic anti-inflammatory effect when combined with other substances. Zheng et al. [17] found that the best synergistic effects were observed under the combined conditions of silymarin (8 uM) and CAP (16 uM), and this integration was reported to be beneficial in restraining the generation of NO, TNF-α, IL-6, and cyclooxygenase-2 (COX-2) caused by LPS. Oner et al. [18] found, in the rhinitis model treated with CAP, that CAP combined with steroids could lower the levels of cytokines (IL-4, IL-5, IL-13, and IL-33), along with the counts of eosinophils and basophils, and had a therapeutic effect on experimental allergic rhinitis. The anti-inflammatory mechanism of CAP is shown in Figure 2. In summary, this suggests that CAP has a good anti-inflammatory effect and the combination of CAP with other substances can reduce inflammation more effectively. The anti-inflammatory effects of CAP are outlined in Table 1.

### 2.3. Antioxidant Activity

Oxidative stress is triggered when oxidative reactions become unbalanced in an organism, leading to the generation of more oxidized molecules and free radicals than the body can effectively eliminate. One of the possible shared etiologies of a number of cardiovascular illnesses is increased oxidative stress.

Some particular components in chili peppers can function as dietary antioxidants, such as stimulating capsaicinoids. Some studies showed that CAP in the concentration range of 5–25 μM had a protective effect on the mitochondrial membrane of rat liver, which could reduce the radiation-induced formation of thiobarbituric acid-reactive substances (TBARS) and lipid hydroperoxides, and the protective effect increased linearly with the increase of the CAP concentration [21]. Li et al. [22] realized that dietary CAP supplementation elevated uncoupling protein 2 (UCP2) and nuclear factor erythroid 2-associated factor 2 (Nrf2) downstream antioxidant enzyme genes through the TRPV1/protein kinase A/UCP2 signaling pathway, thus improving jejunum redox dysfunction induced by heat stress in mice. Thus, the oxidative harm to the small intestine of the affected mice was somewhat alleviated.

By increasing glutathione (GSH) levels, reactive oxygen species (ROS), malondialdehyde, plant cysteine oxidase, and certain oxidized protein derivatives, CAP has a strong protective impact against oxidative stress [23]. An earlier study revealed that CAP had the ability to regulate antioxidant activity by promoting the upregulation of Nrf2 protein levels and concurrently increasing serum concentrations of catalase (CAT) and superoxide dismutase (SOD) [24]. The mechanism of the antioxidative process highlights the role of CAP in promoting the antioxidant reaction without changing its fundamental significance. CAP has good antioxidant properties; nevertheless, there are few investigations on the antioxidant properties of CAP analogues, and researchers are still exploring the antioxidant properties of CAP analogues. The antioxidant effects of CAP are shown in Table 2.

### 2.4. Antimicrobial Activity

CAP shows antibacterial characteristics against a range of microbes and is frequently utilized in agriculture, the food sector, chemical engineering, and medicine. At present, research has demonstrated that CAP may hinder the development of several microorganisms, such as *Staphylococcus aureus*, Group A hemolytic *Streptococci*, *Enterococcus species*, and *Listeria monocytogenes* [27]. Capsaicinoids can bind to the cell wall via lipid interactions that break down the peptidoglycan structure and make the cell membrane more fluid [28]. This makes it easier for ions (K^+^ and Ca^2+^) and CAP to enter the cytoplasm. Osmotic stress is created when the solute enters the cell, leading to increased water absorption and the promotion of cell lysis. In addition, CAP disrupts the expression of genes involved in the development and reproduction of microorganisms, which eventually results in modifications to growth rates and suppression of microbes.

It has been demonstrated that CAP decreases *Helicobacter* counts at doses of more than 10 μg/mL, which lowers the likelihood of illnesses brought on by these bacteria in a dose-dependent way [29]. CAP also showed good antimicrobial properties in combination with other active ingredients. Zhai et al. [30] added CAP to the alkaline electrolyte to trigger the development of ZnO nanopillars and then obtained ZnO/Zn nanopillar films via electrodeposition. When the electrolyte included 0.6 g/L of CAP, regular ZnO nanopillars formed. The generated ZnO/Zn nanopillar films demonstrated strong antibacterial activity in *Escherichia coli* samples with minimal live bacteria coverage. CAP not only has antibacterial activity but also has certain inhibitory effects on some fungal cells. Behbehani et al. [31] tested the susceptibility of CAP to *Candida* spp. using the CLSI method. The results showed that CAP inhibited ergosterol biosynthesis in the cell wall and greatly decreased the mature biofilm of *Candida albicans* by 70% to 89%. However, ergosterol was used by fungi to maintain cellular integrity, membrane fluidity, and cellular metabolism, and inhibiting its biosynthesis disrupted the structure and integrity of the cell [31]. In summary, the primary mechanism by which CAP inhibited microbial growth and reproduction was by damaging the integrity of microbial cell membranes, which allowed cellular contents to seep out. CAP may also inhibit the growth of microorganisms by affecting their metabolic pathways and gene expression, and the specific mechanisms need to be further explored.

### 2.5. Anticancer Activity

Cancer remains one of the most prevalent causes of mortality around the globe. Ten million deaths are thought to have been caused by this illness annually [32]. Therefore, it is necessary to take certain measures to curb cancer, and in recent years, CAP has been found to perform well in alleviating cancer. In liver cancer cells, CAP treatment downregulated the expression of silent information regulator 1 (SIRT1), resulting in reduced SOX2 deacetylation, thereby reducing the stability of SOX2 and leading to the transfer of SOX2 from the nucleus to the cytoplasm. Reduced SOX2 levels inhibited the dryness of liver progenitor cells, thereby inhibiting the further progression of hepatocellular carcinoma [33]. In hepatocytes, by activating the SIRT1/NOX4 signaling pathway, CAP caused the anti-apoptotic protein Bcl-2 to decrease, the pro-apoptotic protein Bax to increase, and the apoptosis-related proteins CASP3 and CYC to increase [34]. This ultimately caused the death of liver cancer cells and successfully stopped the further progression of liver cancer. It has been proven that CAP alters the expression of the cyclin-dependent kinase 8 (CDK8) protein within the breast carcinoma cells, resulting in the cells entering a G2/M arrest during the cell cycle. In addition, the downregulation of CDK8 also affected the phosphate inosine 3 kinase (PI3K)/protein kinase B (Akt) regulatory pathway, thereby inhibiting the β-catenin signaling pathway and influencing the proliferation and movement of tumor cells [35].

An essential transcription factor that controls tumor metabolism in hypoxic environments is hypoxia inducible factor (HIF-1α). Under hypoxic circumstances, researchers discovered that CAP decreased target genes’ expression and HIF-1α protein accumulation and inhibited mitochondrial respiration to increase intracellular oxygen levels [36]. CAP is an agonist for TRPV1 and has been shown to be an anticancer agent by impeding cell proliferation and encouraging apoptosis across various types of cancer cells. In undifferentiated thyroid carcinoma 8505C cells, CAP might directly disturb intracellular calcium homeostasis by activating TRPV1 channels, which caused mitochondrial calcium overload. Moreover, excessive calcium effects triggered mitochondrial permeability transition pore opening, mitochondrial malfunction, and finally, cell demise [37]. Research has demonstrated that CAP triggers death by regulating the expression of proteins like survivin and protein phosphatase 1 (Ki-67), which are linked to both apoptosis and cell proliferation, as well as by downregulating the proto-oncogene FBI-1, which inhibits the activation of the NF-κB signaling pathway [38]. These effects have been demonstrated in many types of cancers, including oral, thyroid, and breast cancers, and so on (Table 3).

## 3. Impacts of CAP on In Vivo Metabolism

### 3.1. Impacts of CAP on Lipid Metabolic Pathways

By promoting lipolysis and oxidation and preventing lipid production and absorption, CAP controls lipid metabolism [40]. It has been found that CAP could regulate the bile acid composition, activate the lipid X receptor, inhibit the manifestation of fibroblast growth factor 15 (Fgf15), and promote the level of expression of protein cholesterol 7α-hydroxylase (CYP7A1), thus resulting in regulating lipid metabolism and reducing the levels of triglyceride (TG) and total cholesterol (TC) [41]. It was found that in HepG2 cells, CAP could reduce circadian asymmetry and inhibit OA-induced overproduction of ROS and mitochondrial dysfunction, while it was less effective in mitigating the effects of OA-induced lipid accumulation when the Bmal1 gene was downregulated [42]. Bort et al. [43] also found that CAP inhibited the AKT/mTOR pathway, activated AMP-activated kinase (AMPK), modulated the activity of peroxisome proliferator-activated receptors, and blocked autophagy. These mechanisms were all employed to explain the anti-lipogenic impact of CAP in HepG2 cells.

**Figure 3:**
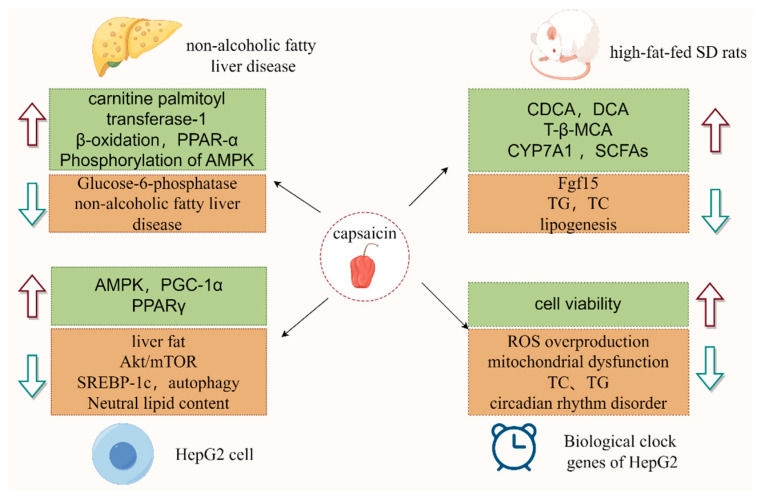
The mechanism by which CAP influences lipid metabolism pathways. In the figure, red indicates promotion and green indicates inhibition.

CAP also acts on brown adipose tissue, which is a highly specialized thermogenic tissue with high metabolic thermogenesis. CAP activated its receptor TRPV1, which activated sympathetically mediated brown adipose tissue and promoted fat burning and energy expenditure, thereby contributing to weight loss and weight control [44]. In addition, numerous metabolically active tissues contain TRPV1, which regulates a number of processes, including the activation of metabolic regulators and adipocyte browning. Takeda and Dai [45] found that in addition to activating UCP-1, CAP could also activate a number of metabolic genes linked to the metabolism of fatty acids, the glycolytic pathway, and adaptive thermogenesis, which promoted the browning of adipocytes and improved the transformation efficiency of brown adipocytes. Through modifications to the makeup of bile acids and reductions in the quantities of some common bile acids, CAP might have an impact on both the conventional and alternative routes of cholesterol conversion. The mechanism by which CAP influences lipid metabolism is depicted in Figure 3. In summary, CAP acts on lipid metabolism through multiple mechanisms and shows potential to ameliorate metabolic abnormalities caused by a high-fat diet.

### 3.2. Effects of CAP on the Mechanisms of Glucose Metabolism Pathways

The primary way that CAP affects glucose metabolism is via reducing the blood sugar concentration. Alkaloids exert their therapeutic effects on the development of blood glucose issues through various signaling circuits and routes. These cascades and pathways include the inhibition or stimulation of numerous systems, including α-glucosidase inhibition, insulin sensitivity augmentation, and oxidative stress management [46].

Zhang et al. [47] found that CAP treatment activated streptozotocin-induced TRPV1 channels in the organs known as the liver and pancreas of diabetic rats, promoted the expression of pancreas and pancreaticoduodenal homology box-1, and elevated the expression of glucose transporter 2 (GLUT2)/glucokinase (GK) and insulin receptor substrate (IRS1/2), promoting insulin secretion and playing a hypoglycemic role. Ultimately, blood glucose levels significantly decreased, while serum insulin, muscle glycogen, and liver glycogen levels increased. Hui et al. [48] examined how CAP regulates the balance of glucose in mice that have type 2 diabetes. They found that CAP had a major impact on the intestinal microbiota’s composition. Specifically, it inhibited the growth of *Lactobacillus* and diminished bile salt hydrolase (BSH) activity, which resulted in the buildup of tauro-β-muricholic acid (TβMCA). Moreover, the increase in TβMCA levels inhibited the gut–liver FXR-Fgf15 signaling pathway and promoted CYP7A1 expression and liver bile acid synthesis, which led to an increase in liver glycogen synthesis and an improvement in glucose homeostasis [48]. Gong et al. [49] also found that CAP could modify the makeup of bile acid by elevating the concentrations of particular bile acids, such as β-muricholic acid, deoxycholic acid, chenodeoxycholic acid, and 3β-ursodeoxycholic acid. These increases activated the Farnesoid X receptor (FXR) and G-protein-coupled bile acid receptor (GPBAR1/TGR5), thereby regulating glucose metabolism and energy metabolism, which in turn regulated glucose metabolism and energy metabolism [50]. Research has demonstrated that CAP improves intestinal flora associated with the generation of endogenous agonists of the TGR5 receptor, such as lithocholic acid, and elevates the emission of glucagon-like peptide-1 (GLP-1), which is found in L cells in the intestinal tract. It also strengthens insulin secretion through the GLP-1 receptor, which improves glucose homeostasis [50].

**Figure 4:**
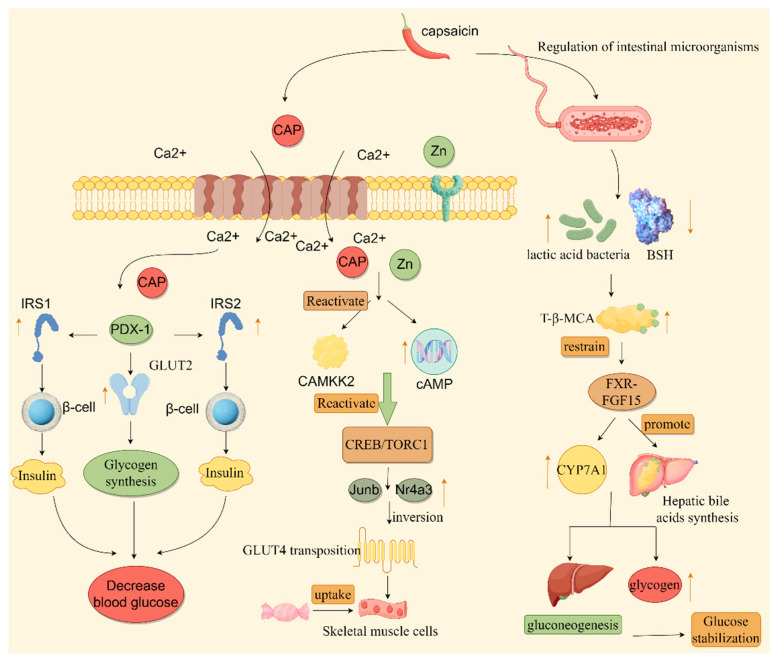
The mechanism through which CAP influences glucose metabolic processes. In this figure, the yellow upward arrow means up-regulation, the yellow downward arrow means down-regulation, and the black arrow indicates the process arrow.

According to recent research, the Zn signaling system is involved in glucose metabolism as well. Ferdowsi et al. [51] found that CAP and Zn treatment activated calcium-signaling-related molecules by increasing the intracellular cytoplasmic calcium concentration, which in turn promoted the expression of downstream genes pertinent to downstream sugar metabolism, specifically involving the upregulation of transcription factors, such as Atf3, Junb, and Nr4a3, thereby promoting glucose transporter type 4 (GLUT4) production and translocation and increasing the absorption of glucose in skeletal muscle cells. In addition, CAP and Zn increased intracellular cAMP levels, and the increase in cAMP also aided in the activation of molecules linked to calcium signaling, which facilitated the absorption of glucose [51]. In insulin-resistant mice, dietary CAP has been demonstrated to boost glucose metabolism in insulin-resistant rats by downregulating inflammatory factors and NF-κB and upregulating GLUT4 expression to open TRPV1 channels [52]. The mechanisms by which CAP affects glucose metabolism are summarized in Figure 4. When combined with other substances, CAP can also be beneficial for the metabolism of glucose. In another study, it turned out that when CAP and metformin (MET) were given to diabetic rats, the combination dramatically decreased fasting blood glucose, enhanced endurance for glucose, relieved inflammatory infiltration and liver damage, downregulated inflammation-related cytokines, and upregulated intestinal tight junction protein [53]. In terms of glucose metabolism, CAP promotes insulin secretion and cellular uptake and utilization of glucose, thereby lowering blood sugar levels [40]. These benefits can effectively prevent the onset of diabetes and a variety of other glucose-related disorders while maintaining blood sugar homeostasis.

### 3.3. Effects on Metabolic Pathways of Intestinal Flora

The “organ” of the human body, the intestinal flora, is essential to the immunological, nutritional, and metabolic systems of the body. Intestinal flora’s content and composition may be impacted by CAP [54]. When CAP was added to mice with diet-induced obesity, the number of bacteria capable of producing butyrate increased and butyrate production was stimulated, which improved the intestinal barrier’s integrity [55]. Cheng et al. [56] found that long-term high-dose ingestion of CAP might alter the makeup and quantity of the gut flora. CAP not only altered the number of bacteria linked to mucus, like *Akkermansia muciniphila* and *Muribaculaceae*, but also changed those associated with bile acid metabolism. In addition, CAP could also activate TRPV1 channels and enhance the expression of mucin 2 and mucin 3 in the mucus layer of the colon, resulting in increased mucus thickness, which provided a suitable growth environment and nutrient source for intestinal probiotics, thereby increasing their abundance [57].

CAP primarily influences the intestinal flora metabolic pathway by encouraging the creation of short-chain fatty acids (SCFAs). Some organic acids, such as acetate, which are metabolites of the gut flora, are collectively known as SCFAs and are essential for gut health and overall metabolism. Santos et al. [58] found that CAP augmented the proliferation of advantageous gut microbiota, including those that make SCFAs. Furthermore, the expansion of the beneficial flora encouraged the synthesis of SCFAs, which helped to better maintain the integrity and health of the intestinal tract. In addition, CAP enhances the secretion level of lgA in the stool and promotes intestinal health. Clinical research has demonstrated that CAP preserves intestinal integrity by altering the properties of the gut flora, modifies the gut–brain axis to modulate hunger–satiety hormones, and prevents obesity by influencing energy metabolism [59]. It can be seen from the above that CAP has great potential in promoting intestinal flora, and some CAP-based functional foods can be developed in the future to better promote intestinal health.

## 4. Novel Delivery Systems for CAP

**Figure 5:**
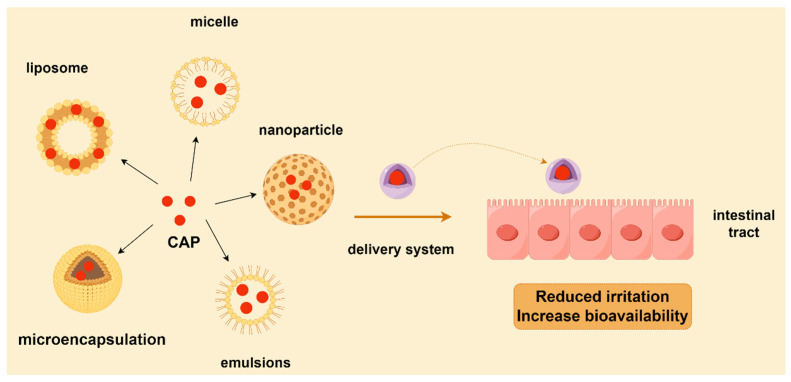
Delivery systems for CAP.

The biological activities and pharmacological effects of CAP are diverse. However, the primary drawbacks of CAP stem from its brief half-life and categorization within the Biopharmaceutical Classification System as Class II, due to its limited bioavailability [60]. Moreover, CAP may cause irritation to the oral mucosa and gastrointestinal tract when ingested in high concentrations or in large amounts, causing discomfort or even side effects. Improving bioavailability can reduce the total intake of CAP while guaranteeing efficacy, thereby reducing the risk of its potential side effects. The bioavailability of CAP may be impacted by its low water solubility and stability. In response to these problems, scientists have created a range of advanced delivery technologies (such as liposomes and nanoparticles) that can greatly enhance the stability and loading capacity of CAP and raise its bioavailability. Several types of delivery systems to enhance the bioavailability of CAP will be described below (Figure 5).

### 4.1. Nanoliposomes

Nanoliposomes are nanoscale bilayer membrane structures composed of lipid materials, such as phospholipids and cholesterol. The structural characteristic of liposomes is a bilayer spherical structure that is spontaneously formed by amphiphilic molecules, characterized by the hydrophilic heads oriented toward the external aqueous surroundings and the hydrophobic hydrocarbon chains located inside [61]. Liposomes are the perfect medication carriers for many polar compounds due to their amphiphilicity. Due to their excellent drug encapsulation ability, large specific surface area, tiny particle size, superior stability, and good biocompatibility, nanoliposomes have a wide variety of application prospects in the biomedical sectors, including vaccine administration, gene therapy, and drug delivery systems. In addition to hydrophobicity and hydrophilicity, nanoliposomes can also enhance the solubility, stability, and biological availability of some unstable bioactive substances that are not fully utilized.

Some studies show that the pharmacokinetic properties of CAP can be enhanced by loading CAP molecules into a nanoliposome model and testing their anticancer potential. At a dosage of 1 mg/kg, the administration of CAP via lipid multi-particulate (LMP) formulations markedly increased the bioavailability of CAP in the serum of rats [62]. Using nanoliposomes can also enhance the biological availability of CAP in the bodies of people. The stimulation of 0.25% CAP nanostructured lipid carriers (NLCs) on human skin was studied. The results showed that capsicum-extract-loaded NLCs were suitable for transdermal administration with minimal skin irritation and less skin irritation compared to CAP alone [63]. NLC formulations were more effective in terms of encapsulation efficiency, in vitro CAP release, and permeability properties compared to solid lipid nanoparticle (SLN) formulations, thereby enhancing the delivery of CAP to the dermal layer of the skin [64]. NLCs enriched with cayenne pepper extracts showed promise in delivering high doses of CAP via the skin. Additionally, in vivo experiments on skin irritation showed that conventional CAP patches resulted in skin irritation and redness, but patches with CAP-loaded NLCs had less adverse effects on the skin [65]. In conclusion, the loading of CAP and nanoliposomes can well improve the bioavailability of CAP.

### 4.2. Nanoparticles

Nanotechnology methods are currently gaining popularity worldwide because of the extraordinary physical, chemical, and biological properties of nanoparticles, which have a size range of 1 to 100 nm. Nanoparticles can be synthesized or loaded with plant molecules, both of which boost the potential for the particles and the loaded portion to come into play. Additionally, they can significantly lessen the side effects of the substance by increasing their solubility in water, which lowers the overall dosage required.

In a study, calcium carbonate was used as a drug carrier, and this carrier was used to encapsulate CAP to form morphologically stable CaCO_3_-loaded CAP nanoparticles, which were coated with a phospholipid bilayer and polyethylene glycol and were also well dispersed and stabilized in aqueous solution [66]. Potentially useful in treating tumors, the released CAP could efficiently activate the TRPV1 channel, causing a dramatic rise in the intracellular calcium ion concentration and subsequent cell death [66]. Abulencia et al. [67] prepared CAP nanovesicles with an encapsulation effectiveness of 92.3% and an average diameter of 134 nm by using a modified thin-film hydration process. Similarly, using an improved duck egg chorioallantois membrane experiment, the researchers found that encapsulating CAP in nanovesicles was able to minimize its stimulation by nearly 50 percent and increase anti-inflammatory activity by 10 times [67]. Zhu et al. [68] investigated the effect of hyaluronic acid (HA)/polylactic acid (PLA) composite nanoparticles with different composite ratios using CAP as a model drug on drug release and bioavailability. The results showed that HA/PLA composite nanoparticles could increase CAP solubility and demonstrate good long-term controlled release, which would increase the drug bioavailability in vivo [68]. It can be seen that CAP-loaded nanoparticles did not merely elevate the bioavailability of CAP, they also somewhat lessened the discomfort that CAP causes.

### 4.3. Emulsions

Emulsions are a common carrier system for encapsulating and delivering hydrophobic active ingredients, such as CAP. Emulsion loading technology effectively protects the encapsulated CAP from degradation, oxidation, or inactivation during storage, processing, or transportation. Additionally, the emulsion loading technology can also increase the contact area of the CAP with the organism by dispersing it in an emulsion, thereby increasing its solubility and bioavailability [69]. Nanoemulsions are similarly capable of improving the bioavailability of lipophilic compounds by increasing the interfacial area [70]. Several emulsions are now available for loading CAP, increasing its solubility and reducing the irritation it causes.

In one study, sodium alginate was combined with CAP to create a water-soluble CAP dispersion system, and then the CAP was mixed with corn oil, beeswax, and polyglycerol ricinoleate under high-speed shear conditions to obtain a water-in-oil high internal phase emulsion (W/O HIPE) loaded with CAP. The encapsulation rate of CAP was 98.7%, and the emulsion remained stable after 28 days of storage [71]. In in vitro digestion simulation experiments, CAP-loaded W/O HIPE showed little CAP leakage from the emulsion under simulated oral and gastric conditions, and the slow and sustained discharge of CAP in an imitation intestinal fluid experiment effectively improved its bioavailability. Luo et al. [72] found that Tween 80 emulsions encapsulating CAP possessed a soft gel-like texture, which facilitated simpler release of oil droplets from the protein matrix, thereby promoting the liberation of CAP molecules during intestinal digestion. Furthermore, Tween 80 molecules that were moved away from the interface potentially contributed to the creation of mixed micelles, which aided in the dissolution and release of CAP molecules and might improve their bioavailability. The stabilized CAP-based nanoemulsions could be developed using solvent substitution methods. The findings of cytotoxicity assays revealed that this nanoemulsion lessened the harmful effects of CAP on cells, hence reducing the adverse effects of CAP, including irritation, poor water solubility, and high levels of cytotoxicity [73]. In one study, CAP was encapsulated in an oil-in-water double emulsion system with ethanol. The CAP-loaded emulsion preserved its compartmental structure to prevent CAP leaking for months after mimicking oral and gastric digestion. In addition, the small intestine digested this emulsion, releasing CAP and greatly increasing the biological accessibility of CAP capsules [74]. Emulsion systems have the potential to improve the biological acceptability of CAP and reduce its irritating properties. Through carefully designed emulsion formulations and preparation methods, effective encapsulation and controlled release of CAP can be achieved, which has been applied in food, nutraceuticals, and pharmaceuticals.

### 4.4. Micelles and Microcapsules

Micelles are nanoscale structures formed by amphiphilic polymer molecules in aqueous solution through a self-assembly process. These polymer molecules possess hydrophilic heads and hydrophobic tails, which spontaneously arrange themselves in water to form aggregates with specific structures [75]. This unique structure makes micelles ideal carriers for hydrophobic food functional components and can effectively improve the solubility and bioavailability of these components in aqueous environments. Bao et al. [76] synthesized CAP-containing α-lactalbumin nanomicelles and utilized microneedle patch technology to administer medication. CAP, kaempferol, retinol, genistein, curcumin, and other hydrophobic bioactive substances may all be effectively bound by α-lactalbumin. Under acidic conditions, this micelle preferentially releases CAP in adipose tissue. Research conducted on DIO mice showed that CAP was efficiently delivered by the microneedle patch to abdominal fat tissue, where it was taken up by white adipocytes and caused a marked reduction in body weight. Its mode of action was linked to enhanced mitochondrial biogenesis, adipocyte browning induction, and energy metabolism activation. Zuo et al. [77] demonstrated that nanomicelles overcame the p-glycoprotein efflux pump function, which stopped CAP from being removed from the cell, and dramatically enhanced cellular anti-lipogenesis. They also accelerated endocytosis of CAP. In addition, in comparison to free CAP, the CAP-loaded nanomicelles markedly reduced adipogenesis and increased lipid hydrolysis and macroscopic autophagy. It is well known that its use in food is further limited due to its strong irritation and gastrointestinal irritation. Enveloping CAP in nanomicelles produced from partial hydrolyzed α-lactalbumin increased its water solubility and bioavailability considerably [78]. Such nanomicelles showed good colloidal stability in vivo and were able to mitigate the irritating taste of CAP, making it more widely used in food applications. The aforementioned research has demonstrated that the efficacy and variety of applications of CAP can be greatly increased by combining it with micelles.

Solid or liquid nuclear material can be enclosed in microcapsules, which are microcontainers with an organic or inorganic shell made of a core-shell structure [79]. The process of encapsulating small substances in a hermetically sealed or semi-permeable capsule membrane using natural or synthetic polymers to create a solid, stable product is known as microencapsulation [80]. Encapsulation could significantly improve the solubility of CAP by selecting a suitable shell material and preparation process. In addition, the encapsulated CAP could be better absorbed by the gastrointestinal tract, thus improving its bioavailability and enhancing the efficacy of CAP by improving the solubility and controlling the release to achieve the same or a better therapeutic effect at a lower dose. Kulig et al. [81] used CAP solution and alginate oligomers to create binuclear capsules. The results showed that the alginate oligomer could be used for the encapsulation of CAP with an encapsulation efficiency of more than 80%, which improved the antioxidant performance of the end products, imparted its capacity to reduce iron ions, and improved the durability of CAP under stomach conditions. In one study, chitosan and carboxymethyl chitosan were used as the shell materials to create CAP microcapsules via a layer-by-layer self-assembly technique. After encapsulation, CAP was suddenly released in three solutions within seven days, and then slowly released. The release performance was highest in alkaline solution, followed by acidic and neutral salt solution [82]. In other research, whey protein and modified starch were mixed with octenyl succinic anhydride to create spray-dried CAP microcapsules. The increase in the whey protein concentration increased the yield of the final product and augmented its encapsulation efficiency, wettability, and dissolution rate, whereas microencapsulation dramatically increased the CAP stability and water solubility [83]. A tiny quantity of octenyl succinic anhydride, a modified starch combined with whey protein, is a potential CAP carrier that might help advance the use of CAP in food. The application of microencapsulated CAP can greatly overcome some of the limitations of CAP in applications and maximize the bioavailability of CAP. These technologies provide effective ways to apply CAP in food and medicine.

### 4.5. Other Delivery Systems

Apart from the above delivery systems, a number of other delivery systems are crucial in improving the bioavailability of CAP. Some studies focused on complex-loaded CAPs to investigate their role in improving CAP solubility and bioavailability. In a study, cricket protein isolates and alginate complexes were employed to encapsulate CAP. The complex created by this encapsulation system had greater antioxidant activity than the unencapsulated CAP, according to quantitative analysis, which also indicated that the CAP was well encapsulated in this complex and that an effective release of CAP was achieved with a 91% encapsulation efficiency [84]. The hydrophobic interactions between oleic acid and CAP enable the effective incorporation of CAP into the hydrophobic structural regions of the chitosan–oleic acid composite, thereby successfully constructing composite materials encapsulating CAP by combining the ethanol solution containing CAP with the aqueous solution of chitosan [85]. Throughout the duration of the investigation, the complex particles were stable and could be kept at room temperature for longer than three months [85]. Guo et al. [86] prepared a protein–rhamnose–lipid complex loaded with CAP, and they produced a complex with good physical and thermal stability by changing the mixing ratio of pea protein isolate/whey protein isolate with CAP. As a consequence, CAP was well encapsulated in the complex, which better improved its water dispersion and thus significantly improved the bioavailability of CAP. Overall, although CAP has some drawbacks, the emergence of the new delivery system well compensates for the drawbacks of CAP, which could better promote the application of CAP in food.

## 5. Various Applications for CAP

### 5.1. Application of CAP for Food Preservation

As a physiologically active ingredient in chili peppers with low content but great value, CAP not only has a significant antibacterial effect, but also can effectively fight against oxidation, and thus can slow down the process of food spoilage. In the field of food preservation, CAP has also been innovatively applied to active packaging technology, which significantly extends the food shelf life through the combination of advanced materials to prepare a slow-release antibacterial and antioxidant function of the preservation film. Furthermore, food can be treated directly with CAP to improve its fresh flavor and prevent bacterial development without affecting the color and flavor of the product. Therefore, CAP shows a broad application prospect in food preservation and injects new vitality into the continued success of the food processing sector.

#### 5.1.1. CAP Is Applied as a Preservative and Antioxidant for Food Preservation

With the rapid development of modern society, the food industry, while bringing convenience and abundant choices, is also faced with the challenge of how to preserve nutrients and flavor while extending the shelf life. Traditional synthetic antioxidants and preservatives, while meeting this need to a certain extent, have become a growing concern for consumers because of their potential safety hazards and health risks. In response to this problem, a number of natural antioxidants and preservatives are being developed to provide health benefits while serving to preserve food. These naturally occurring substances, which include sugar, salt, acetic acid, and some plant-based active compounds (like CAP, essential oils, etc.), work to keep food fresher for longer by preventing bacteria from growing and reproducing. Compared to synthetic preservatives and antioxidants, they are kinder to the human body and reduce the health risks that may be associated with long-term intake of these substances. Therefore, natural antioxidants have a promising application, usually including some plant extracts, animal extracts, and some metabolites [87]. CAP is one kind of natural antioxidant, and it is a good choice to add CAP in food.

Mariadoss et al. [88] prewashed uniformly sliced fresh-cut *Capsicum annuum* (FCCa) with sodium hypochlorite solution, then applied a 10% concentration of *Capsicum annuum* ethanol extract (CAE). They then inoculated the surfaces of the FCCa with two foodborne pathogens, *Listeria* and *Salmonella enterica*, before preserving them for 12 days at 15 °C and 4 °C. The results showed that combined treatment with sodium hypochlorite and CAE could considerably decrease the pathogen load on peppers, maintain the color of pepper, improve its antioxidant status, and successfully prolong the duration of storage of FCCa kept at 4 °C [88]. In addition to the role of CAP in the above foods, it can also replace chemical preservatives in meat products to play a preservative role.

It is well known that nitrites and nitrates are preservatives frequently used in meat products. They are essential for preventing microbial development and regulating the oxidation of fat. However, while they provide delicious flavor, they also pose certain risks to our health. Therefore, the use of nitrites and nitrates in cured meat products needs to be replaced or reduced. For example, the addition of pepper/chili powder nanoparticles to cured meats and meat products during processing can optimize curing and improve consumer acceptance [89]. Jeong et al. [90] incorporated 0.7 mg of sodium nitrite and 5 mg of paprika powder extract to pork patties, individually. After 14 days of storage, meat patties treated with chili powder extract and sodium nitrite had comparatively low pH values and cooking losses, while the levels of lipid and protein oxidation in the patties were also lower, effectively inhibiting spoilage and ensuring the quality of the products. The effectiveness of paprika extract in avoiding pH variations during storage, as well as in inhibiting protein and lipid oxidation and minimizing cooking loss, was comparable to that of sodium nitrite [90]. Therefore, chili pepper extract can replace sodium nitrite as a natural preservative in foods. Studies have shown that compared with the group without added cayenne pepper, emulsified sausage with 3% yellow cayenne pepper powder added had a significantly higher water retention capacity and cooking rate, as well as the emulsified sausage’s volatile alkali nitrogen levels and thiobarbituric acid-reactive components were all noticeably decreased [91]. The emulsified pork sausage’s antioxidant activity was considerably enhanced, and its preservation performance was enhanced by the addition of 3% yellow pepper powder. Moreover, the role of CAP extends beyond the quality enhancement of pork meat products, as it also exhibits a significant preservative effect on beef and duck meat products. Zaher et al. [92] added red pepper extract to ground beef. Due to the biological composition, autolytic enzymes, microbial activity, and lipid oxidation of minced meat, its shelf life was relatively short, while the addition of red pepper extract to ground beef could prolong its shelf life by preventing the growth of foodborne illnesses and spoiling microorganisms [92]. Moreover, cooked meatballs made from ground beef with paprika extract were not distinct significantly from those without paprika extract with respect to flavor, tenderness, juiciness, and overall acceptability, and they had a longer shelf life and lower levels of *Enterobacter* and *Staphylococcus aureus* contamination [92]. It is commonly recognized that the color of meat products is very important, and it directly determines the consumer’s desire to purchase the meat products. When duck flesh is roasted at high temperatures, myoglobin is often over-oxidized, which might affect the meat’s color [93]. Wu et al. [93] added CAP and dihydrocapsaicin to myoglobin isolated from duck meat and found that CAP and dihydrocapsaicin could bind to myoglobin, improving its oxidative stability in duck meat. This suggests that CAP and dihydrocapsaicin may be useful natural antioxidants for enhancing meat products’ color. From the above, it can be seen that the usage of CAP as a preservative and antioxidant in food has significant advantages and broad prospects, and most of the application of CAP is for meat products, which is good to promote the development of meat products.

#### 5.1.2. CAP as a Plant Active Ingredient Added to Food Packaging Materials for Food Preservation

As can be seen from the above, attempts are already being made to incorporate plant active ingredients into food products to give them better color, quality, and superior shelf life. And of course, there are researchers who are already favoring the use of plant-based actives to develop food packaging. It is well known that plastics have been used far more frequently in the food packaging sector, however, only a tiny fraction of plastic bags are degraded, which poses a significant threat to the environment. So, there is a significant rise in consumer needs for edible and natural food packaging. Edible coatings and films not only operate as a part of the food, providing nutrition to the body, but also have the important task of protecting the food, slowing down its deterioration, and thus significantly extending its shelf life [94]. These coatings or films are enriched with natural plant extracts, which effectively retard or stop the growth of microbes, ensuring that the quality of the food is maintained.

Su et al. [95] fabricated ethyl cellulose composite films containing CAP. The results showed that the resulting ethyl cellulose–CAP films were not only transparent and soft but also had waterproof and environmentally friendly properties. What is more, this composite film exhibited significant bacteriostatic activity. In addition, the freshness retention effect of the composite film was also tested on bell peppers. The test findings demonstrated that the freshness of bell peppers with the ethyl cellulose–CAP films were well maintained after one week of preservation compared to unpacked bell peppers that were prone to shrinkage and decay [95]. There are also applications in fruits. Zhao et al. [96] fabricated a gelatin and chitosan composite film using a hollow metal–organic framework loaded with CAP as an antimicrobial biomaterial for food packaging. The phase separation issue was successfully resolved by using the fe^III^-doped hollow metal–organic framework as a CAP nanocarrier, which could control the hydrophobicity of CAP. They covered fresh, white, and highly hydrated apple slices with laminated film. After five days of storage, they observed that the surface of the unwrapped apple pieces gradually darkened after one, three, and five days. On the 5th day, under ideal circumstances, the gelatin/chitosan film wrapped around the apple pieces effectively reduced moisture loss, controlled oxidation, and no bacterial growth was observed, maintaining good freshness [96]. However, the application of CAP in the packaging industry also targets meat products. Poultry and beef products have become much more popular in recent years. However, owing to the easy growth of microorganisms in poultry meat, it is susceptible to oxidative spoilage. Consequently, for a longer shelf life of fresh poultry, the meat processing sector must employ the proper preservation processes. Rather et al. [97] preserved chicken breasts in gelatin packages using nano-emulsified chili seed oil. The chicken breast pieces packed in these bags had less UV transmittance and a well-controlled color change after 15 days of storage. Additionally, microbiological analysis revealed that the samples effectively suppressed microbial development, with *E. coli* levels remaining below acceptable limits. More importantly, the packed samples had slowly increased pH values and low thiobarbituric acid reactant values [97]. The packaging material retarded lipid oxidation and meat spoilage. As can be seen from the above, CAP, a plant active ingredient, plays a role in different aspects of food preservation; however, in fact, CAP blended into food packaging to preserve food research is relatively small, and it is hoped that in the future there can be more research to deepen this aspect, to develop more food preservation methods.

#### 5.1.3. Preparation of Functional Foods

It is evident from the foregoing that CAP has strong antibacterial action against viruses and foodborne pathogens, as well as strong antioxidant activity in food matrices [98]. These properties make it valuable in the creation of functional foods that more than improve the sensory experience of the food but additionally provide the customer with extra health advantages.

Research has been performed on creating fortified yogurt by incorporating various concentrated sweet pepper extracts (CSPE) into the yogurt recipe. After 21 days of refrigeration, the fortified CSPE yogurt had an irregular surface, a tighter texture, and a good orange color. Not only was it higher in hardness and consistency than other yogurts, but it was also rich in probiotics, which better promote digestion [99]. Goktas et al. [100] attempted to develop functional chocolates with antioxidant activity by adding CAP extract (PE) to white compound chocolate and chocolate sauce because these two chocolates are free of cocoa solids, and they lack antioxidant activity. Before and after in vitro digestion, antioxidant activity was significantly elevated when PE was used to produce white compound chocolate and chocolate sauce samples [100]. Kim [101] developed chicken sausage with added pepper seed to reduce the total fat, saturated fatty acid, and cholesterol content of chicken sausage by replacing chicken skin with chili seeds. CAP, bioactive, and pungent components were present in the pepper seed so that the sausage had good looks, flavor, and general appeal, in addition to a decent level of stiffness [101]. The addition of chili seeds to chicken sausage boosts the food’s nutritional content and offers a healthy substitute for those who like low-fat cuisine. Avci et al. [102] used a by-product derived from hot pepper seed oil to devise a low-fat salad dressing. The incorporation of this by-product significantly improved the pseudoplasticity, viscoelastic characteristics, and the ability to recover of the low-fat salad dressing, resulting in excellent rheological attributes and enhanced oxidative stability. Although not much research has been done on functional foods with CAP, with the continuous pursuit of healthy foods by consumers, functional foods with CAP are expected to become a bright star in the food industry, bringing more healthy choices to consumers around the world.

### 5.2. Applications of CAP in Combating Challenging Diseases

CAP has a variety of biological activities, thus making it potentially therapeutic for diseases, such as cancer. CAP has anticancer effects on different types of cancers, such as stomach, cervical, prostate, colon, nasopharyngeal, lung, esophageal, interstitial thyroid degeneration, glioblastoma, kidney, and breast cancers [103,104,105]. Andretta et al. [106] demonstrated that CAP suppresses the growth of both parental and cisplatin-resistant mesothelioma cells by inducing S-phase cell cycle arrest and inhibiting the lateral movement and migration of mesothelioma cells. Vel et al. [107] treated lung cancer cell lines with 25–150 μM of CAP. It was found that 101.2 μM CAP-treated cells significantly reduced their viability, the number of cells was also reduced, and the number of apoptotic cells increased [107]. CAP significantly promoted apoptosis and inhibited cancer cell migration. In addition to having applications in the fight against cancer, CAP can also alleviate the symptoms of severe viral infections. Strengthening the immune system is a key point in targeting viral infections. TRPV-1 has been described as a potential target in autoimmune diseases and as a modulator of neuroinflammation. Janda and Yadarola [108] showed that TRPV-1 receptors play an important role in the prognosis of very specific viral infections, such as COVID-19. Numerous studies have shown that 3CL-protease and RNA-dependent RNA polymerase are responsible for the replication of viral proteins and that inhibition of these enzymes prevents viral replication in the human body [109]. Cortés-Ferré et al. [110] demonstrated that CAP acts similarly to TRPV-1 agonists and can be used as an inhibitor of 3CL-protease and/or RNA-dependent RNA polymerase, thus treating the disease or controlling further spread of the virus. Nahama et al. [111] also showed that interfering with TRPV-1 signaling may reduce the severity of acute respiratory distress syndrome in COVID-19 patients.

## 6. The Challenges Faced by CAP

CAP exhibits sensitivity to various environmental factors, including light, oxygen, acid, alkali, and high temperatures. These properties render it unstable during application, thereby posing a significant challenge in enhancing its stability. Furthermore, CAP’s limited water solubility, pungent taste, and relatively low bioavailability restrict its applicability within the food industry. Excessive consumption of CAP may induce gastrointestinal discomfort, leading to potential adverse effects. Consequently, determining the optimal dosage is crucial to maximize the therapeutic benefits of CAP while minimizing associated discomfort.

## 7. Conclusions and Future Prospects

An in-depth analysis of CAP’s bioactivities, which include analgesic, anti-inflammatory, antioxidant, anticarcinogenic, and bacteriostatic effects, was presented in this paper, along with a summary of some of the uses of CAP as a biologically active component. By influencing certain specific signaling pathways, as well as the presentation of certain genes and proteins, CAP affects metabolism in humans, particularly lipid metabolism, glucose metabolism, and intestinal flora metabolic pathways. A better understanding of CAP’s effects on the metabolic pathways is important for maintaining the body’s health. The multiple delivery systems of CAP effectively increase the bioavailability of CAP and alleviate the burning sensation associated with CAP, thus allowing it to be delivered efficiently and effectively. In addition, CAP has been utilized in food preservation to prevent germ contamination, extend the duration of storage, and conserve food because of its antibacterial and antioxidant qualities. Although CAP has potential uses in food preservation, there is little research on its application in functional food development. More innovative delivery methods based on CAP for active substances and food preservation ought to be built in the future.

## Figures and Tables

**Table 1:** Anti-inflammatory impacts of CAP.

Model Organism	Treatment	Results	References
BALB/c mice	Mouse peritoneal macrophages were isolated and activated for 24 h with 1, 2, 5, or 100 μg/mL of CAP and 1 μg/mL of LPS.	Decreased release cytokines that were conductive to the inflammatory response, including IL-6, TNF-α, and NO.	[14]
IPEC-J2 cell line	After 2 weeks of culturing IPECJ2 cells, they were incubated with different concentrations of CAP ranging from 0 to 300 uM for 24 h.	Reduced LPS-induced protein expression of extracellular signaling-related kinase 1/2 and p65.	[15]
Human Umbilical Vein Endothelial Cells (HUVECs)	For 30 min, HUVECs were exposed to CAP or dihydrocapsaicin at several doses (0, 5, 25, and 50 µM), respectively.	Reduced gene expression and secretion of pro-inflammatory cytokines induced by LPS via the TLR4/NF-κB signaling pathway.	[16]
RAW264.7 cells	Cultivated RAW264.7 cells for 24 h, then treated with different concentrations of silymarin and CAP.	Nutrient transport protein mRNA abundance was upregulated (e.g., Na+/glucose cotransporter 1).	[17]
Male Wistar	For seven days, intraperitoneal injections of CAP (50 mg/kg) and steroids (10 mg/kg) were given to rats.	Mitigated the activation of NF-κB and its molecular targets in endothelial cells mediated by TNF-α.	[18]
Human-induced pluripotent stem cell (iPSC)-derived cardiomyocytes (hiPSC-CM)	Incubated cells with media containing the agonist CAP (10 μM).	Significantly reduced monocytes’ adherence to the surface of endothelial cells.	[19]
Male and female rat pups, five days old (P5)	Intraperitoneal injections of 0.2, 1, and 5 mg/kg of CAP were given to P5 rat pups.	Induced NO production.	[20]

**Table 2:** Antioxidant effects of CAP.

Model Organism	CAP Dosage	Mechanism of Action	References
Wistar female rats, aged three to four months	5–50 µM	Shielded the antioxidant enzyme SOD from oxidative damage brought on by radiation. Inhibition of the endogenous antioxidant GSH depletion caused by radiation.	[21]
Male C57BL/6 J mice	0.4 mg/day for 15 days	Enhancing the antioxidant capacity of the intestine via the pathways TRPV1/PKA/UCP2 and Keap1/Nrf2.	[22]
Male adult (8 weeks old) Wistar rats	2 mg/k bw	Enhancement of Nrf2 protein expression and antioxidant activity of SOD and CAT enzymes in the serum.	[24]
Female Sprague–Dawley rats	150–1500 µg/k bw	Increased hepatic GSH values. Reduced serum nitric oxide and protected the liver and lungs from LPS-induced tissue damage.	[25]
Male Kunming mice	7.5 mg/k bw	Reduced malondialdehyde levels and increased expression of glutathione peroxidase, SOD, and CAT in the liver.	[26]

**Table 3:** Anticancer effects of CAP.

Model Organism	Treatment	Mechanism of Action	Results	References
Diethylnitrosamine-induced hepatocellular carcinoma model in rats	Low-dose group: 1 mg/kg liposomal CAP twice a week High dose group: 2 mg/kg liposomal CAP, subcutaneous injection for 4–6 weeks.	Hepatocellular carcinogenesis was decreased when the SIRT1/SOX2 signaling pathway was used to inhibit the stemness of HPCs.	Reduced SIRT1 and SOX2 protein levels. Apoptosis in HepG2 cells.	[33]
Cell line MDA-MB-231	Cells were grown at distinct CAP doses (0, 10, 50, 100, and 200 uM) and at 37 °C with 5% CO_2_ for 48 h.	Reduced CDK8 expression and caused G2/M cell cycle arrest through the restraint of the Wnt/β-catenin/PI3K/CDK8/Akt signaling path.	G2/M cell cycle blocked. The levels of CDK8, PI3K, and Akt expression were lowered. Wnt and β-conjugated protein expression was downregulated.	[35]
A549 cell line H1299 cell line H2009 cell line H23 cell line	Injections of CAP (20, 50, or 100 µM) treatments.	Inhibiting the mitochondrial respiration of lung cancer cells reduced ATP production and the accumulation of HIF-1α, thereby suppressing cancer cell proliferation.	Inhibited the buildup of HIF-1α protein. ATP synthesis in cellular mitochondria was reduced and, therefore, cancer cell growth was inhibited.	[36]
Anaplastic thyroid cancer cell 8505C	Cells received treatment with 50–200 uM CAP for 24 h.	Through a TRPV1-mediated mechanism, CAP caused mitochondrial calcium excess and death in anaplastic thyroid carcinoma (ATC) cells.	Mesenchymal thyroid cancer cells exhibited both apoptosis and mitochondrial calcium excess.	[37]
Human breast cancer cell lines (MCF-7 and MDA-MB-231)	After 24 h of cell attachment culture, treat with different concentrations of CAP.	By suppressing the NF-kB signaling pathway mediated by FBI-1, CAP impeded the growth of breast cancer cells.	Decreased expression of the proteins FBI-1, Ki-67, Bcl-2, and survivin. Inhibited cell proliferation.	[38]
Syrian hamsters	Control group: oral cancer was triggered by DMBA alone, excluding CAP. Experimental group: tumor induction with dimethylbenzanthracene followed by CAP application in the digestive tract.	Reduced the expression of the proteins Bcl-2 and ki-67, which in turn triggered apoptosis and stopped the development of cancer cells.	Encourage the death of malignant cells.	[39]

## Abbreviations

The following abbreviations are used in this manuscript:

Akt	protein kinase B
AMPK	AMP-activated protein kinase
Bcl-2	B-cell lymphoma-2
Bax	Bcl-2-associated X-protein
BSH	bile salt hydrolase
COX-2	cyclooxygenase-2
CAT	catalase
CDK8	cyclin-dependent kinase 8
Cyt c	cytochrome C
CYP7A1	protein cholesterol 7α-hydroxylase
CDCA	chenodeoxycholic acid
CAMKK2	calmodulin-dependent protein kinase kinase 2
cAMP	cyclic adenosine monophosphate
DCA	deoxycholic acid
FBI-1	human immunodeficiency virus-1
Fgf15	fibroblast growth factor 15
GSH	glutathione
GLUT1/2/4	glucose transporter 1/2/4
GLP-1	glucagon-like peptide-1
IL-4/5/6/13/13	interleukin-4/5/6/13/33
IL-1β	interleukin-1β
IRS1/2	insulin receptor substrate 1/2
ICAM-1	intercellular cell adhesion molecule-1
Junb	Jun B proto-oncogene
Ki-67	proliferation-related protein
LPS	lipopolysaccharide
mTOR	mammalian target of rapamycin
mPTP	mitochondrial permeability transition pore
NF-kB	nuclear factor-kappa B
Nrf2	NF-E2-related factor 2
NOX4	NADPH oxidase 4
Nr4a3	Nuclear Receptor Subfamily 4 Group A Member 3
PI3K	phosphate inosine 3 kinase
PDX-1	pancreatic duodenal homeobox 1
PPAR-a/γ	peroxisome proliferator-activated receptor a/γ
PGC-1a	peroxisome proliferator-activated receptor gamma coactivator-1 alpha
ROS	reactive oxygen species
SOD	superoxide dismutase
SIRT1	silent information regulator 1
SREBP-1c	sterol regulatory element binding protein-1c
SCFAs	short-chain fatty acids
TNF-a	tumor necrosis factor-alpha
TLR4	toll-like receptor 4
TRPV1	transient receptor potential cation channel subfamily V member 1
TC	total cholesterol
TG	triglyceride
T-β-MCA	tauro-β-muricholic acid
UCP2	uncoupling protein 2
VCAM-1	vascular cell adhesion molecule-1
